# Supplementary material for: Structure and thermodynamics of water adsorption in NU-1500-Cr
Source: Commun Chem. 2023 Apr 15;6:70. doi: 10.1038/s42004-023-00870-0 (PMC10105746; doi:10.1038/s42004-023-00870-0)
Supplement: Supplementary file 2 — Supplementary Information [file 42004_2023_870_MOESM2_ESM.pdf]

# Supplementary Information

# Structure and thermodynamics of water adsorption in NU-1500-Cr

C.-H. Ho et al.

**Supplementary Figures**

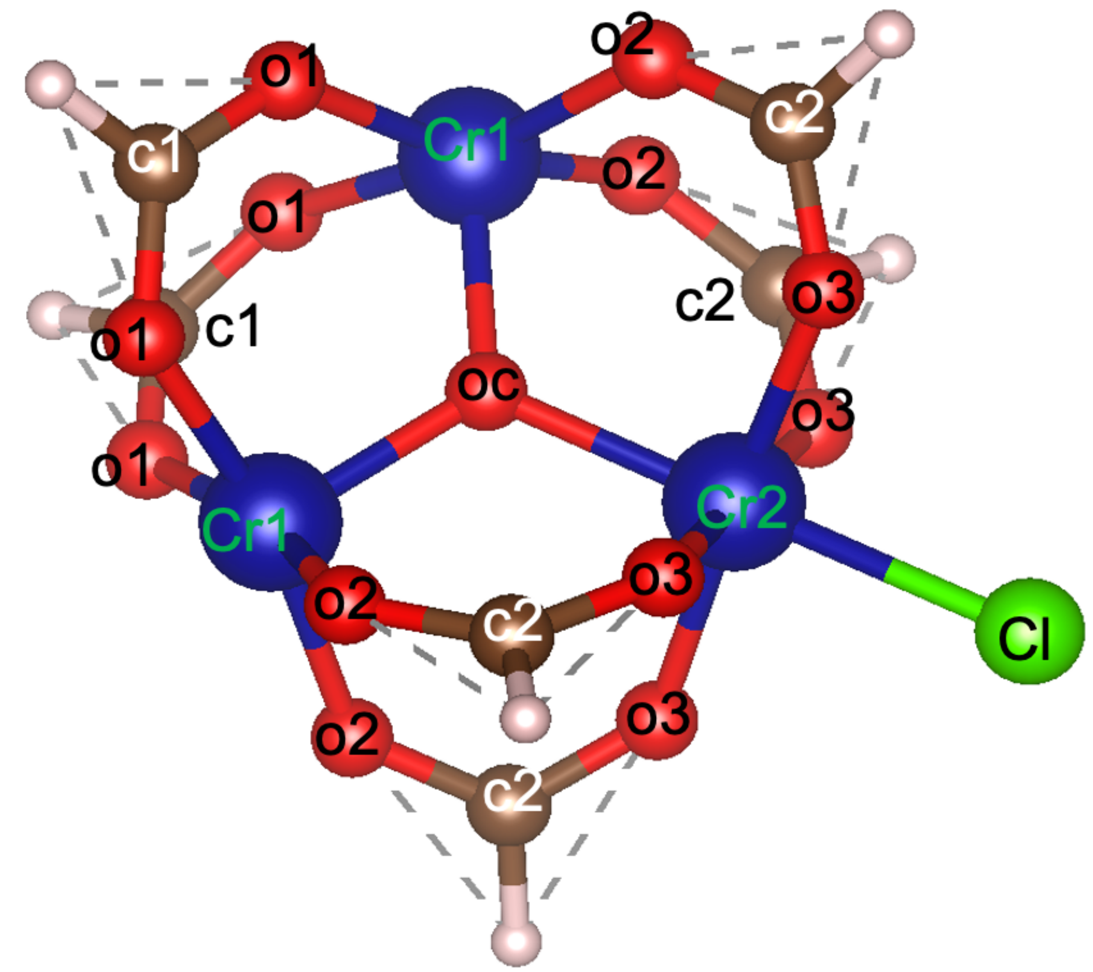


**Supplementary Figure 1**: **Force field development for NU-1500-Cr.** Atom labels used in the force field parameters for the secondary building unit (SBU) of NU-1500-Cr.

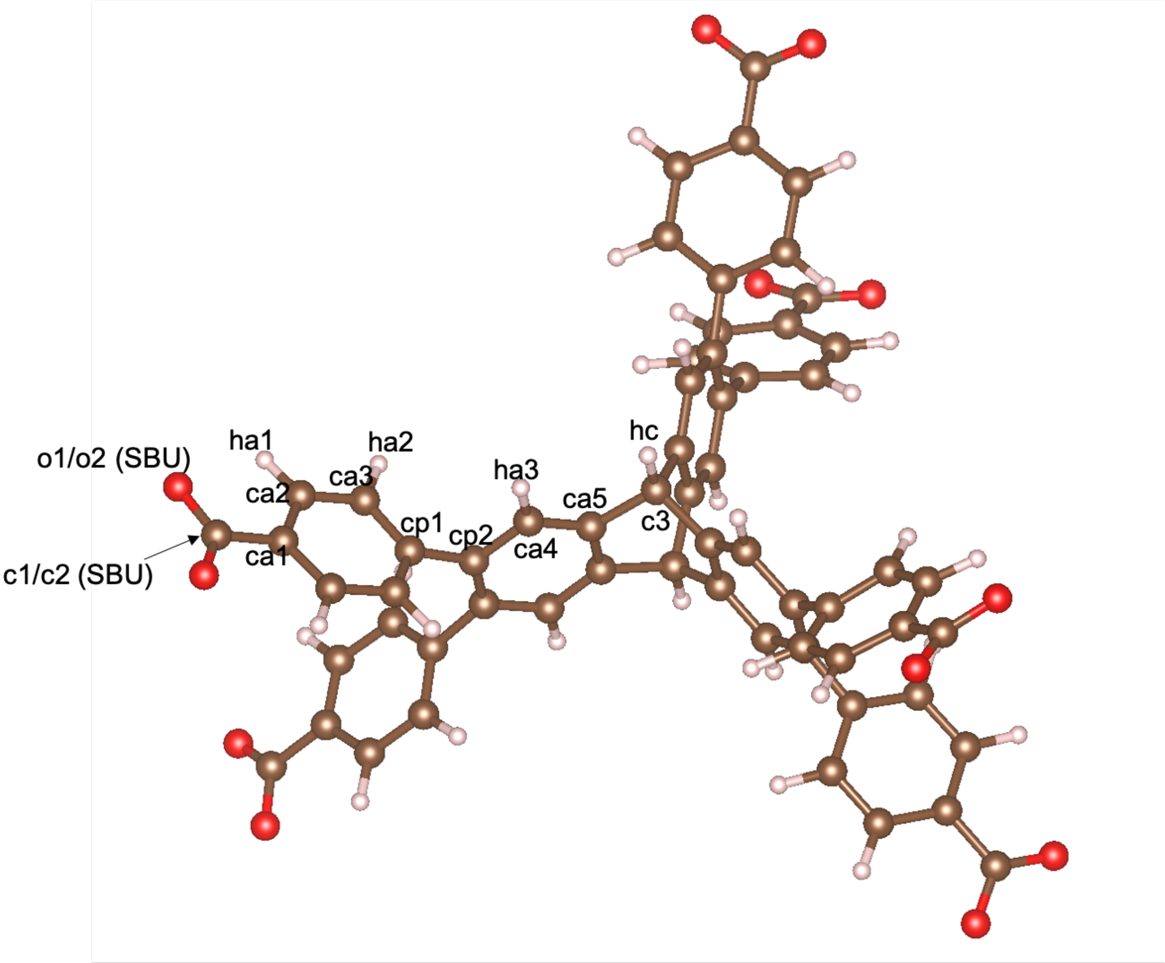


**Supplementary Figure 2**: **Force field development for NU-1500-Cr.** Atom labels used in the force field parameters for the organic linker, deprotonated hexacarboxylic acid peripherally extended triptycene (PET), in NU-1500-Cr. For clarity, the definitions are only labeled on one of the branches. Unlabeled atoms can be inferred through the symmetry of the molecule.

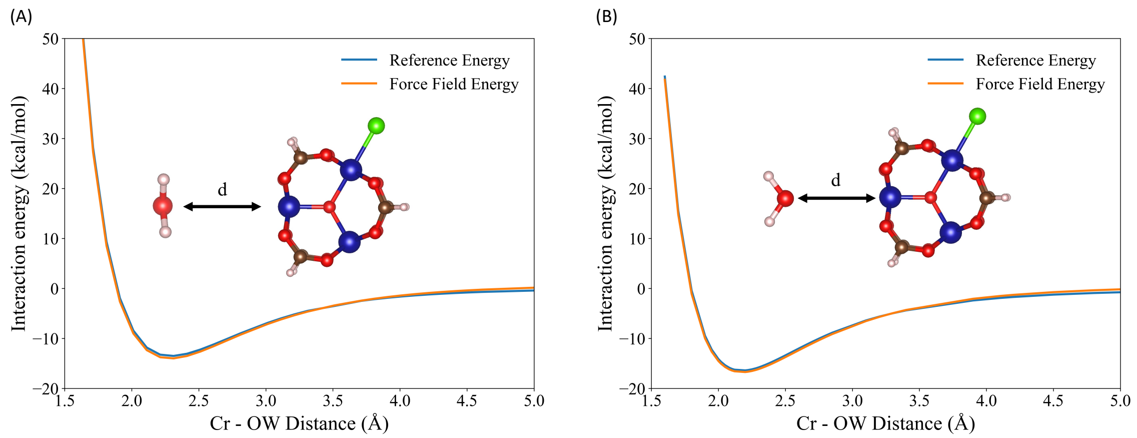


**Supplementary Figure 3**: **Potential energy scans of water-SBU complexes with (A) parallel (B) perpendicular orientations relative to the SBU.** Water–MOF interactions are parameterized using Lorentz-Berthelot mixing rules using the Lennard-Jones (LJ) values for the MOF listed in Supplementary Table 1 and for the TIP4P/2005 water model, except for the atom pairs listed in Supplementary Table 5 whose interactions were directly fitted to *ab initio* water–SBU potential energy scans.

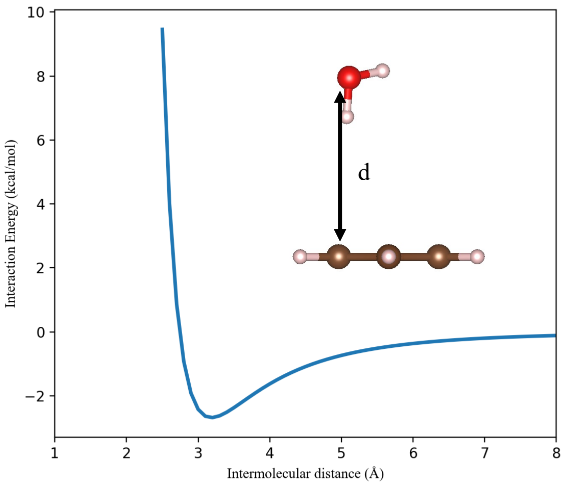


**Supplementary Figure 4**: **Water–benzene potential energy scan.** Interaction energy between $H_2O$ and benzene calculated using the parameters listed in Supplementary Table 1. The CM5 charges were calculated using Gaussian 16 with the same functional and basis set discussed in the main text. One of the OH bonds of the water molecule points toward the aromatic ring of benzene. The intermolecular distances are defined along a vertical line perpendicular to the plane of benzene.

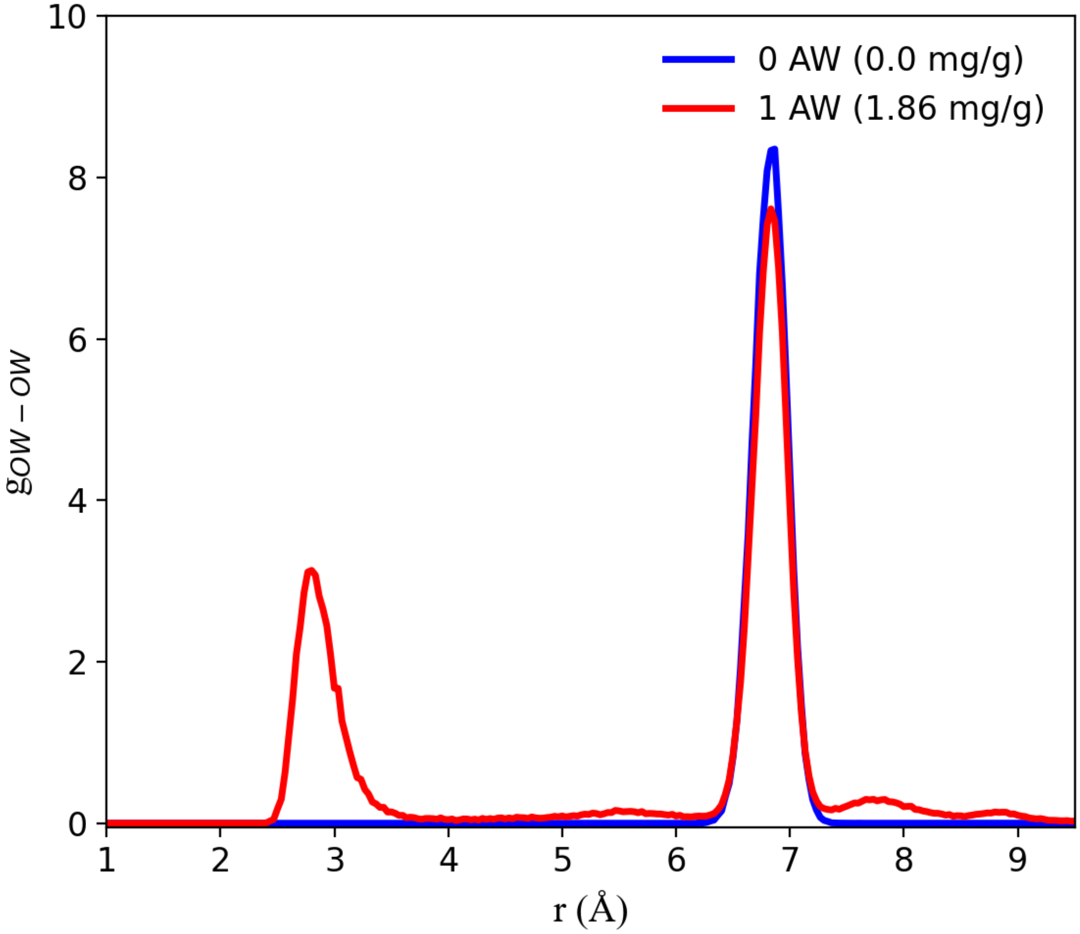


**Supplementary Figure 5**: **Oxygen-oxygen radial distribution functions.** Oxygen-oxygen radial distribution functions calculated for NU-1500-Cr with each $Cr^{3+}$ site saturated by one water molecule (blue) and for the same system with an additional water molecule (red).

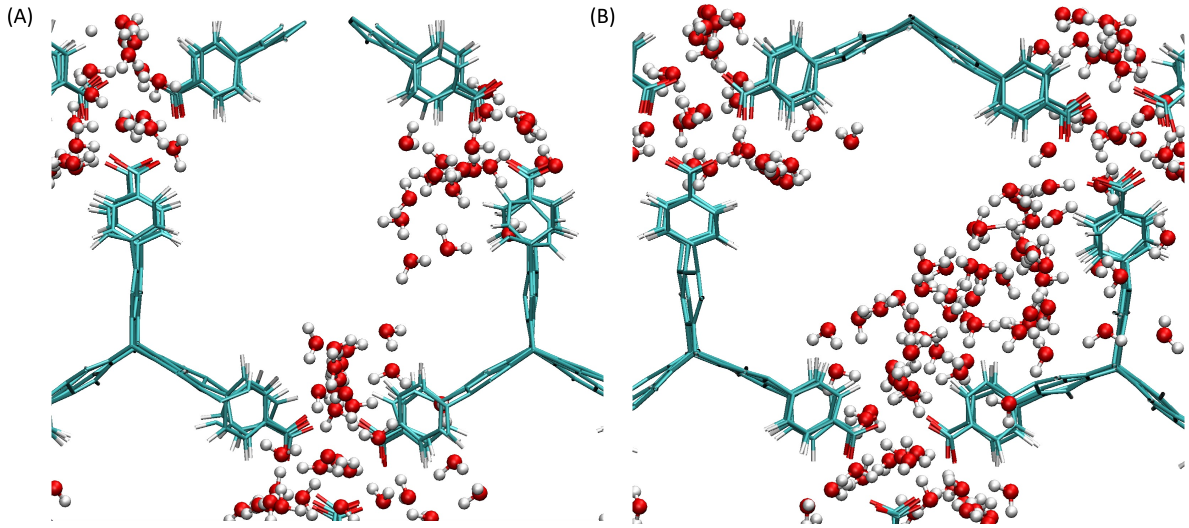


**Supplementary Figure 6**: **Snapshots of main pores filling.** Snapshots of the water distribution at (A) 33.0% RH (B) 33.5% RH.

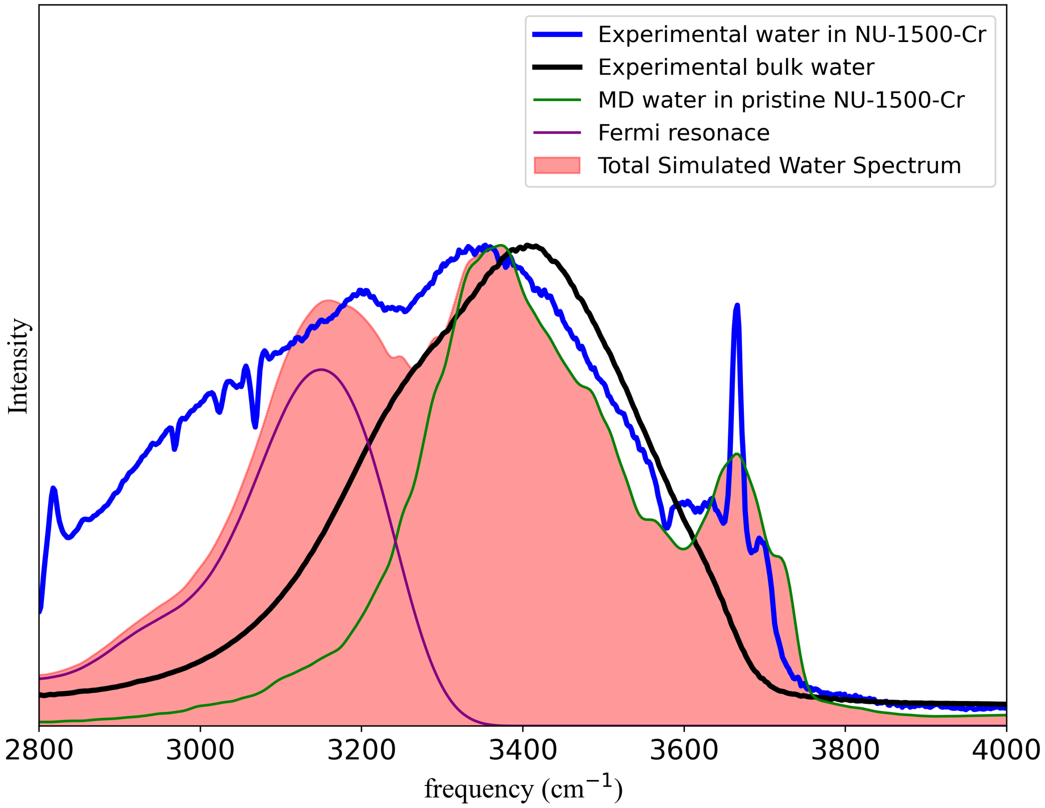


**Supplementary Figure 7**: **Experimental and simulated OH-stretch bands of water.** Blue: Experimental spectrum of water in NU-1500-Cr at 25% RH. Black: Experimental spectrum of bulk water. Green: Simulated spectrum of water in NU-1500-Cr at 25% RH. Purple: Simulated bending overtone (Fermi resonance) of water in NU-1500-Cr at 25% RH. Shaded red area: Total simulated spectrum of water in NU-1500-Cr.

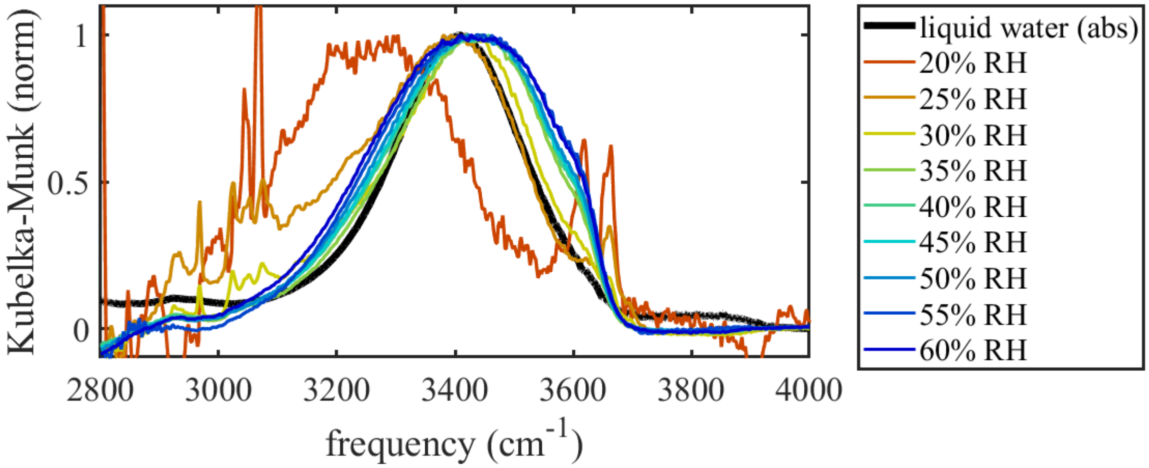


**Supplementary Figure 8**: **OH-stretch band of HOD in NU-1500-Cr compared to the OH-stretch band of HOD in bulk water.** The OH-stretch bands are shown for HOD in bulk $D_2O$ (black) and for HOD in $D_2O$-filled pores of NU-1500-Cr at RH 20% and 60% RH. The low-frequency tail is particularly prominent for spectra at 20% (red) and 25% (orange) RH.

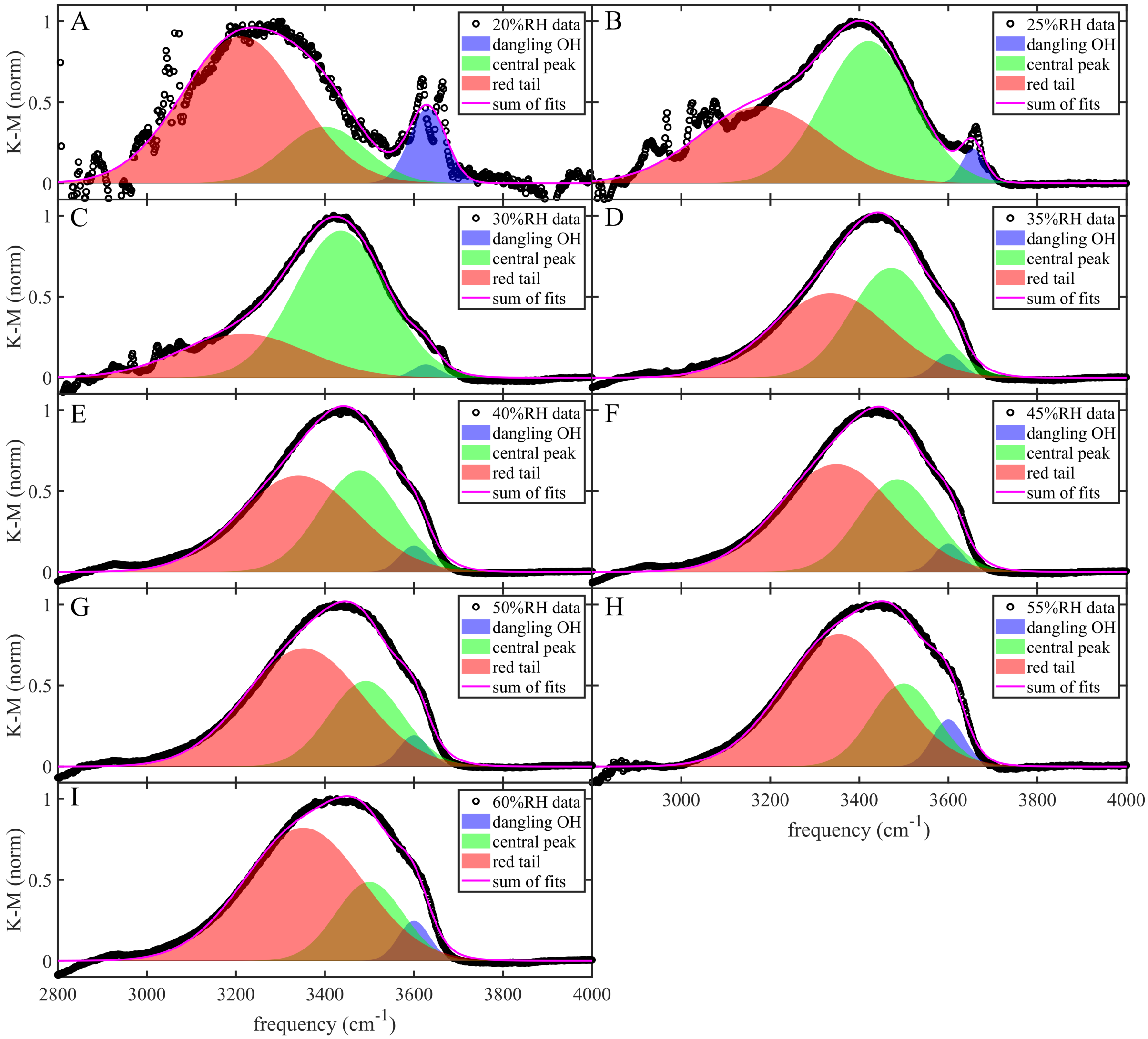


**Supplementary Figure 9**: **Gaussian fits to the OH-stretch band of HOD in NU-1500-Cr.** The spectra were fit at (A) 20% RH, (B) 25% RH, (C) 30% RH, (D) 35% RH, (E) 40% RH, (F) 45% RH, (G) 50% RH, (H) 55% RH, and (I) 60% RH. The red tail at 3200 $cm^{-1}$ decreases in intensity from 20 to 30% RH. Another redshifted peak centered around 3300 $cm^{-1}$ is observed at higher humidities.

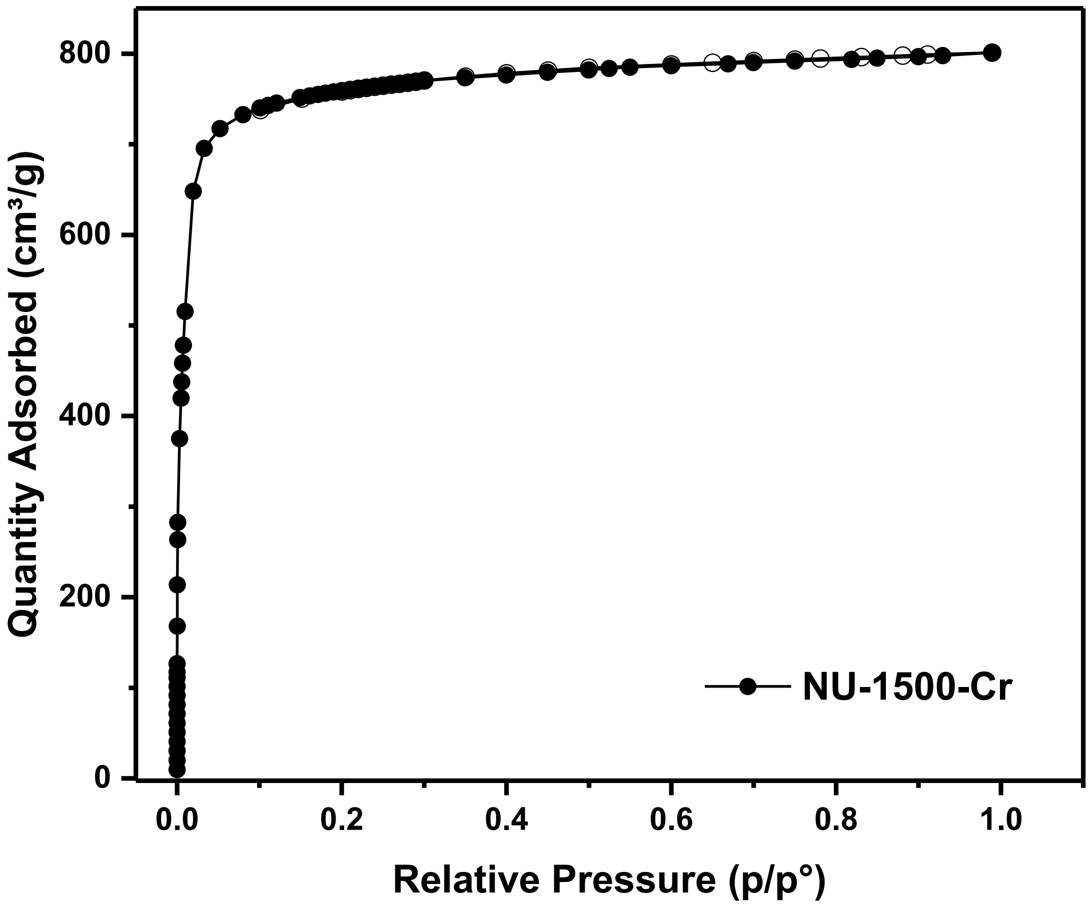


**Supplementary Figure 10**: **$N_2$ adsorption isotherm.** The $N_2$ adsorption isotherm was measured on a Micromeritics ASAP 2420 (Micromeritics, Norcross, GA) instrument at 77 K.

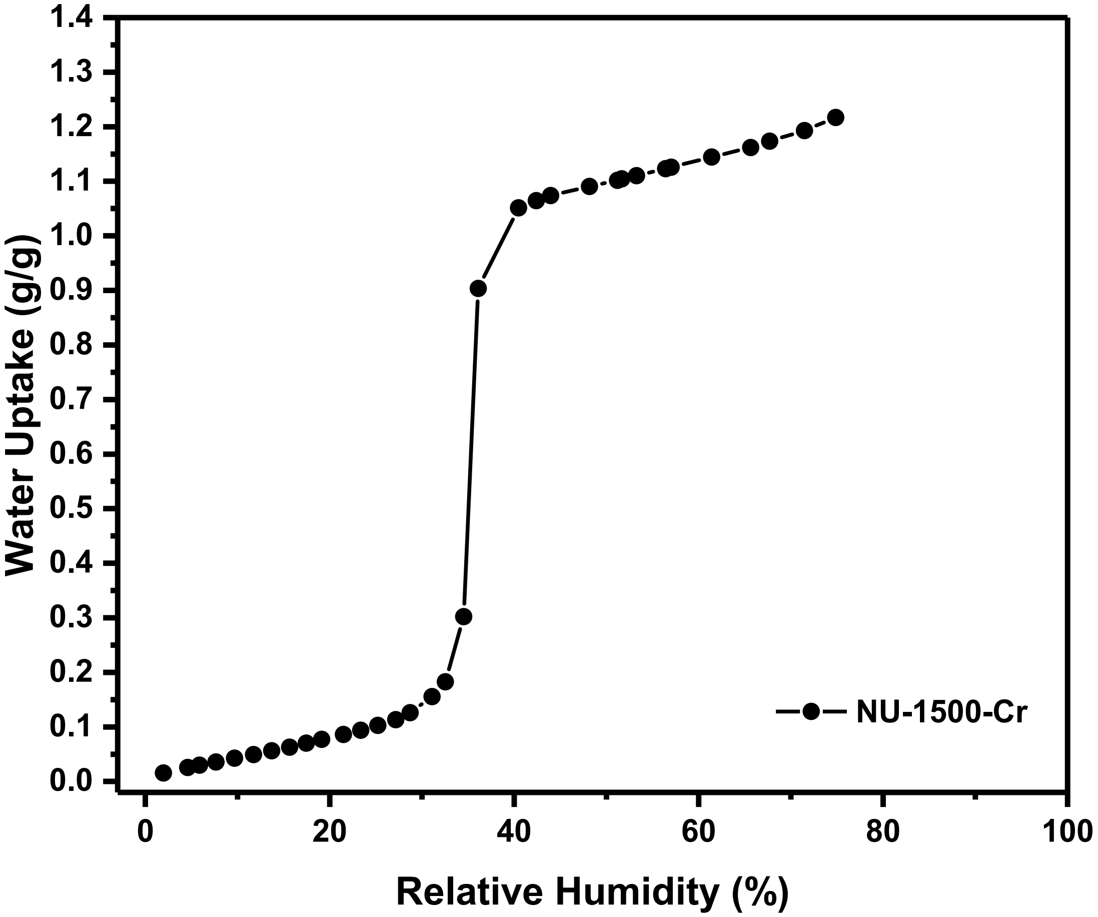


**Supplementary Figure 11**: **Water adsorption isotherm.** The water adsorption isotherm of NU-1500-Cr was measured on a micromeritics 3Flex at 25 °C. It should be noted that the adsorption isotherm shown in Figure 1A slightly differs from the adsorption isotherm reported in ref 1. The difference is due to the use of an incorrect value of the saturated vapor pressure of water at 298 K in the original study, which results to the original adsorption isotherm being shifted to slightly higher RH values relative the adsorption isotherm measured in the present study.

## Supplementary Tables

**Supplementary Table 1**: Force field parameters for NU-1500-Cr: Partial charges and Lennard-Jones parameters.

| Atom name | Atom type | Charge | $\epsilon$ (kcal · mol$^{-1}$) | R/2 (Å) |
|---|---|---|---|---|
| Cr1 | Cr1 | 1.214625 | 0.0150 | 1.5115 |
| Cr2 | Cr2 | 1.112858 | 0.0150 | 1.5115 |
| c1 | c | 0.227263 | 0.0860 | 1.9080 |
| c2 | c | 0.224305 | 0.0860 | 1.9080 |
| ca1 | ca | -0.011949 | 0.0860 | 1.9080 |
| ca2 | ca | -0.069163 | 0.0860 | 1.9080 |
| ca3 | ca | -0.089312 | 0.0860 | 1.9080 |
| ca4 | ca | -0.089361 | 0.0860 | 1.9080 |
| ca5 | ca | -0.008693 | 0.0860 | 1.9080 |
| cp1 | cp | 0.004217 | 0.0860 | 1.9080 |
| cp2 | cp | -0.002250 | 0.0860 | 1.9080 |
| c3 | c3 | -0.057928 | 0.1094 | 1.9080 |
| o1 | o | -0.381737 | 0.2100 | 1.6610 |
| o2 | o | -0.397233 | 0.2100 | 1.6610 |
| o3 | o | -0.360480 | 0.2100 | 1.6610 |
| oc | oc | -0.693786 | 0.2100 | 1.6610 |
| Cl | Cl | -0.467782 | 0.2270 | 1.9735 |
| hc | hc | 0.101268 | 0.0157 | 1.4870 |
| ha1 | ha | 0.116697 | 0.0150 | 1.4590 |
| ha2 | ha | 0.103653 | 0.0150 | 1.4590 |
| ha3 | ha | 0.107425 | 0.0150 | 1.4590 |

**Supplementary Table 2**: Force field parameters for NU-1500-Cr: Bond potentials, $U(r) = \frac{1}{2}K_{ij}(r - r_0)^2$.

| Bond type | $K_{ij}$ (kcal · mol$^{-1}$ · Å$^2$) | $r_0$ (Å) |
|---|---|---|
| Cr1-oc | 430.15 | 1.798 |
| Cr1-o | 342.72 | 1.919 |
| Cr2-oc | 60.010 | 1.702 |
| Cr2-o | 226.77 | 1.800 |
| Cr2-Cl | 158.03 | 2.254 |
| c-ca | 691.80 | 1.491 |
| c-o | 1275.0 | 1.218 |
| ca-ca | 922.20 | 1.398 |
| ca-ha | 691.60 | 1.086 |
| ca-cp | 900.40 | 1.406 |
| cp-cp | 702.80 | 1.485 |
| c3-ca | 642.00 | 1.516 |
| c3-h3 | 661.20 | 1.097 |

**Supplementary Table 3**: Force field parameters for NU-1500-Cr: Angle potentials, $U(\theta) = \frac{1}{2}K_{ijk}(\theta - \theta_0)^2$.

| Angle type | $K_{ijk}$ (kcal $\cdot$ mol$^{-1}$ $\cdot$ rad$^2$) | $\theta$ (°) |
|---|---|---|
| Cr1-oc-Cr1 | 114.82 | 129.5 |
| Cr1-oc-Cr2 | 109.79 | 115.9 |
| Cr1-o-c | 20.986 | 115.1 |
| oc-Cr1-o | 45.174 | 94.93 |
| o-Cr1-o | 20.304 | 157.1 |
| Cr2-o-c | 17.183 | 133.6 |
| oc-Cr2-o | 54.724 | 88.70 |
| oc-Cr2-Cl | 32.998 | 183.0 |
| o-Cr2-o | 31.456 | 182.0 |
| o-Cr2-Cl | 37.314 | 96.97 |
| c-ca-ca | 128.60 | 120.3 |
| ca-c-o | 137.40 | 122.6 |
| ca-ca-ca | 133.20 | 120.0 |
| ca-ca-ha | 96.400 | 119.9 |
| ca-ca-cp | 132.60 | 120.7 |
| ca-cp-ca | 133.40 | 118.4 |
| ca-cp-cp | 128.00 | 121.1 |
| cp-ca-ha | 96.000 | 119.9 |
| cp-cp-cp | 144.80 | 90.00 |
| c3-ca-ca | 127.00 | 120.8 |
| ca-c3-ca | 127.20 | 112.2 |
| ca-c3-hc | 93.600 | 110.5 |
| o-c-o | 155.80 | 130.2 |

**Supplementary Table 4**: Force field parameters for NU-1500-Cr: Torsion potentials, $U(\phi) = K_{ijkl}\left[1+\cos\left(N\phi-\chi\right)\right]$.

| Dihedral type | $K_{ijkl}$ (kcal · mol$^{-1}$) | $\chi$ (°) | N |
|---|---|---|---|
| Cr1-o-c-ca | 5.0040 | 180.00 | 2 |
| Cr1-o-c-o | 3.2376 | 0.00 | 2 |
| oc-Cr1-o-c | 0.6374 | 0.00 | 2 |
| Cr2-o-c-ca | 4.6518 | 180.00 | 2 |
| Cr2-o-c-o | 2.6899 | 0.00 | 2 |
| oc-Cr2-o-c | 2.1991 | 0.00 | 2 |
| Cl-Cr2-o-c | 2.1450 | 180.00 | 2 |
| Cr1-oc-Cr1-o | 0.0000 | 180.00 | 2 |
| Cr1-oc-Cr2-o | 0.0000 | 180.00 | 2 |
| Cr1-oc-Cr2-Cl | 0.0000 | 0.00 | 2 |
| o-Cr1-o-c | 0.0000 | 180.00 | 2 |
| o-Cr1-oc-Cr2 | 0.0000 | 180.00 | 2 |
| o-Cr2-o-c | 0.0000 | 180.00 | 2 |
| c-ca-ca-ca | 3.6250 | 180.00 | 2 |
| c-ca-ca-ha | 3.6250 | 180.00 | 2 |
| ca-ca-ca-cp | 3.6250 | 180.00 | 2 |
| ca-ca-ca-ha | 3.6250 | 180.00 | 2 |
| ca-ca-ca-ca | 3.6250 | 180.00 | 2 |
| ca-ca-cp-ca | 3.6250 | 180.00 | 2 |
| ca-ca-cp-cp | 3.6250 | 180.00 | 2 |
| ca-cp-cp-cp | 1.0000 | 180.00 | 2 |
| ca-cp-cp-ca | 1.0000 | 180.00 | 2 |
| cp-cp-cp-cp | 1.0000 | 180.00 | 2 |
| ha-ca-cp-cp | 3.6250 | 180.00 | 2 |
| c3-ca-ca-cp | 3.6250 | 180.00 | 2 |
| c3-ca-ca-ca | 3.6250 | 180.00 | 2 |
| ca-c3-ca-ca | 0.0000 | 0.00 | 2 |
| hc-c3-ca-ca | 0.0000 | 0.00 | 2 |
| c3-ca-ca-c3 | 3.6250 | 180.00 | 2 |
| cp-ca-ca-ha | 3.6250 | 180.00 | 2 |
| ha-ca-ca-ha | 3.6250 | 180.00 | 2 |
| ha-ca-cp-ca | 3.6250 | 180.00 | 2 |
| c3-ca-ca-ha | 3.6250 | 180.00 | 2 |
| o-c-ca-ca | 1.0000 | 180.00 | 2 |
| c-ca-o-o | 1.1000 | 180.00 | 2 |
| ca-c-ca-ca | 1.1000 | 180.00 | 2 |
| ca-ca-ca-ha | 1.1000 | 180.00 | 2 |
| ca-ca-cp-ha | 1.1000 | 180.00 | 2 |
| cp-ca-ca-cp | 1.1000 | 180.00 | 2 |
| cp-ca-cp-cp | 1.1000 | 180.00 | 2 |
| ca-c3-ca-ca | 1.1000 | 180.00 | 2 |

**Supplementary Table 5**: Force field parameters for NU-1500-Cr: Water–Cr Buckingham potential, $U(r) = \mathrm{A}\exp(-r/\rho) - \mathrm{C}/r^6$.

| Buckingham pair | A | $\rho$ | C |
|---|---|---|---|
| OW-Cr1 | 168233.0 | 0.220 | 1470.0 |
| HW-o | 11292.0 | 0.296 | 597.0 |

**Supplementary Table 6**: Comparison of experimental and simulated lattice parameters.

| | a = b (Å) | c (Å) | Unit cell volume ($Å^3$) | Equilibrium Cr-OW distance (Å) |
|---|---|---|---|---|
| Experiment | 19.503(2) | 11.1225(17) | 3663.8(9) | 2.034(7) |
| Simulation | 19.5025 ± 0.0013 | 11.1222 ± 0.0007 | 3663.58 ± 0.71 | 1.967 |

**Supplementary Table 7**: Partial charges for a modified SBU. All atoms not listed here maintain the same partial charges as in Supplementary Table 1.

| Atom | Charge |
|---|---|
| Cr1 | 1.8219375 |
| Cr2 | 1.669287 |
| o1 | -0.533565125 |
| o2 | -0.549061125 |
| o3 | -0.49958725 |

**Supplementary References**

1. Chen, Z. *et al.* Reticular access to highly porous acs-mofs with rigid trigonal prismatic linkers for water sorption. *J. Am. Chem. Soc.* **141**, 2900–2905 (2019).
